# lncRNA-*PCAT1* rs2632159 polymorphism could be a biomarker for colorectal cancer susceptibility

**DOI:** 10.1042/BSR20190708

**Published:** 2019-07-12

**Authors:** Ming-li Yang, Zhe Huang, Li-na Wu, Rong Wu, Han-xi Ding, Ben-gang Wang

**Affiliations:** 1 The 2nd Oncology Department of Affiliated Shengjing Hospital of China Medical University, Shenyang 110022, China; 2 Genery Surgery Department of Affiliated Shengjing Hospital of China Medical University, Shenyang 110015, China; 3 Tumor Etiology and Screening Department of Cancer Institute and General Surgery, the First Affiliated Hospital of China Medical University, Shenyang 110001, China; 4 Department 1 of General Surgery, the First Hospital of China Medical University, Shenyang 110001, China

**Keywords:** colorectal cancer, PCAT1, Single nucleotide polymorphism, susceptibility

## Abstract

**Background:** Single-nucleotide polymorphisms (SNPs) in lncRNAs could be biomarkers for susceptibility to colorectal cancer (CRC), but the association of *PCAT1* polymorphisms and CRC susceptibility is yet to be studied. **Methods:** Five tagSNPs covering the *PCAT1* gene were detected through Kompetitive Allele-Specific PCR among 436 CRC patients and 510 controls. An expression quantitative trait locus (eQTL) bioinformatic analysis was then performed. **Results:** In the present study, *PCAT1* rs2632159 polymorphism increased CRC risk by 1.37-fold and 2.19-fold in the dominant and recessive models, respectively (*P*=0.040 and 0.041). When the CRC cases were divided into colon cancer and rectal cancer, we found that this polymorphism affected colon cancer risk under the dominant model (*P*=0.022, OR = 1.51) and affected rectal cancer susceptibility under the recessive model (*P*=0.009, OR = 3.03). A more pronounced effect was observed in the male subgroup in that *PCAT1* rs2632159 SNP increased rectal cancer risk by 3.97-fold (*P*=0.017). When *PCAT1* rs2632159 was present, epistatic effects were observed with rs1902432 and rs785005 (*P*=0.011 and 0.008, respectively). eQTL analysis showed that rs2632159 could influence binding with the transcription factors EBF, LUN-1, and TCF12. **Conclusion:** *PCAT1* rs2632159 SNP could be a biomarker for CRC risk. And the rs1902432 SNP might only have potential to be a biomarker for colon cancer risk.

## Introduction

lncRNAs are noncoding RNAs longer than 200 bp without apparent protein-coding potential; they can function as oncogenes or suppressor genes in the process of carcinogenesis [1]. At lncRNA loci, genetic variation can occur, which contributes to a change of lncRNA function. Single-nucleotide polymorphisms (SNPs) are the most common pattern of genetic variation. As reported previously, SNPs have the potential to act as biomarkers for cancer susceptibility [2,3]. Some lncRNA polymorphisms were considered to be predictive of cancer risk, to be useful for early diagnosis, and to aid in the management of personalized therapy [2,4].

The expression patterns of prostate cancer-associated transcripts (PCATs) distinguish benign localized cancer and metastatic cancer samples. In 2012, Prensner et al. [5] reported the discovery of a novel prostate cancer lncRNA, PCAT-1, which alternately demonstrates either repression by PRC2 or an active role in promoting cell proliferation through transcriptional regulation of target genes. Subsequently, Ge et al. [6] identified the up-regulation of PCAT1 expression in colorectal cancer (CRC) tissues, indicating that PCAT1 functions as an oncogene in CRC. Concerning *PCAT1* SNPs, only one investigation has reported their association with cancer susceptibility, which found that *PCAT1* rs1902432 tagSNP was associated with prostate cancer risk [7]. However, no comprehensive analysis of the association of all tagSNPs of *PCAT1* with cancer susceptibility has yet been performed, including for CRC.

In the present study, we thus performed a study to investigate the association between the tagSNPs of the *PCAT1* gene and CRC susceptibility. The present study sought biomarkers predictive of CRC risk and was intended to unearth clues providing a deeper understanding of the etiology of colorectal carcinogenesis.

## Materials and methods

### Patients

This research project was approved by the institutional review board of First Affiliated Hospital of China Medical University. All the participants are according to the principles expressed in the Declaration of Helsinki. A total of 436 CRC patients, who had undergone a surgical operation for CRC at the Shengjing Affiliated Hospital of China Medical University, were consecutively recruited between 2016 and 2017. The presnt study is retrospective and designed as a case-control study. In all of these subjects, the pathological diagnosis was confirmed based on the AJCC classification. According to the site of occurrence, there were 229 colon cancer patients and 207 rectal cancer patients. A total of 510 controls were also recruited from a health check program at the same hospital. And the inclusion and exclusion criteria for the case and control groups were: 1) the race is consistent and are all Asian; 2) the pathological diagnosis is definite clear in case group; 3) the healthy controls have the normal serum biochemical indexes and excluded all other systemic diseases; 4) multiple tumor patients with prostate cancer or bladder cancer or cervical cancer were excluded; and 5) patients who received preoperative radiotherapy or chemotherapy were excluded.

Written informed consent was provided by each individual, and the medical records were used for the pathological diagnosis and TNM staging of the CRC cases. A questionnaire was used to collect information on the smoking, drinking, and family history of the cases. The smoking and drinking information are defined by referring the previously published study [8]: cigarette smoker was defined as who have smoked at least 100 cigarettes during one’s lifetime or had been smoking any amount for at least 1 year, which included current smokers and former smokers. Alcohol drinker was defined as who consumed one or more alcoholic drinks per week for >1 year. One drink equals to one 12-oz can or bottle of beer (5% ethanol), one 5-oz glass of wine (12.5% ethanol), or 1.5-oz measure of liquor (40% ethanol) for each subject.

Whole blood from individuals was collected and blood clots were allowed to form by incubating clot-activating tubes at room temperature for 1 h. Each clot was transferred to a 2-ml centrifuge tube and stored at −80°C until DNA extraction.

### Polymorphisms selected and SNP Genotyping

The polymorphisms selected method was based on our previously published study [9]. The linkage disequilibrium (LD) of *PCAT1* gene was shown in Supplementary Figure 1. Then five SNPs covered *PCAT1* gene (rs1902432, rs4573233, rs710885, rs785005, rs2632159) were selected [8,10–12], other SNPs captured by the selected ones were shown in Supplementary Table S1. Genomic DNA was purified from the blood as described previously [13], with some modifications, and was diluted to working concentrations of 50 ng/l for the *PCAT1* polymorphism genotyping. Polymorphisms-genotyping assay was performed using Kompetitive Allele-Specific PCR (KASP, LGC Genomics, Hoddesdon, U.K.) by Gene Company (Shanghai, China) [14,15] and 10% of the total samples were repeatedly genotyped at the same time for quality control (the concordance rate all reached 100%).

### eQTLs

To perform the promising functional polymorphism of *PCAT1* gene and expression quantitative trait locus (eQTL) analysis, we used the bioinformatical online software Haploreg (http://www.broadinstitute.org/mammals/haploreg/haploreg.php) [16] to mine the data.

### Statistical analysis

In the present study, all analyses were conducted on the CRC risk, colon cancer risk, and rectal cancer risk. The association between lncRNA-*PCAT1* gene polymorphisms and CRC risk was determined using multivariate logistic regression adjusted for age and gender. The lncRNA haplotype was analyzed using the online software SHEsis [17]. The epistatic effects of pairwise interacting factors for the polymorphism of the *PCAT1* gene (rs2632159) with the strongest potential effect and other SNPs on the risk of CRC were analyzed by multivariate logistic regression adjusted for age and gender. The association between the genotype of *PCAT1* SNPs and clinicopathological parameters was calculated using χ^2^ two-sided test.

## Results

### The association of lncRNA-PCAT1 SNPs with CRC susceptibility

In the present study, the *PCAT1* rs2632159 polymorphism was found to increase CRC risk by 1.37-fold and 2.19-fold in the dominant and recessive models, respectively (*P*=0.040 and 0.041). When the CRC cases were divided into colon cancer and rectal cancer, we found that *PCAT1* rs2632159 was associated with colon cancer risk under the dominant model (*P*=0.022, OR = 1.51) and with rectal cancer susceptibility under the recessive model (*P*=0.009, OR = 3.03). In addition, *PCAT1* rs1902432 polymorphism decreased colon cancer risk to 0.59-fold (*P*=0.030, Table 1).

**Table 1:** The association of *PCAT1* polymorphisms and CRC risk

SNP^*^	Genotype	*P*_HWE_	Controls (%)	CRC			CRC vs CON		Colon cancer vs CON		Rectal cancer vs CON	
				All (%)	Colon cancer (%)	Rectal cancer (%)	*P*^†^ value	OR (95% CI)	*P* value	OR (95% CI)	*P* value	OR (95% CI)
rs1902432	TT	0.557	170 (34.07)	160 (36.87)	90 (39.47)	70 (33.98)				1 (Ref)		1 (Ref)
	TC		237 (47.49)	208 (47.92)	111 (48.68)	97 (47.09)	0.830	0.97 (0.71–1.31)	0.638	0.92 (0.64–1.31)	0.874	1.03 (0.71–1.51)
	CC		92 (18.44)	66 (15.21)	27 (11.85)	39 (18.93)	0.163	0.74 (0.49–1.13)	**0.030**	**0.57 (0.33–0.94)**	0.916	0.97 (0.59–1.60)
	CC+TC vs TT						0.519	0.91 (0.6–1.22)	0.252	0.82 (0.58–1.15)	0.893	1.03 (0.72–1.47)
	CC vs TT+TC						0.158	0.76 (0.53–1.11)	**0.030**	**0.59 (0.36–0.95)**	0.897	0.97 (0.63–1.51)
	C vs T						0.229	0.89 (0.73–1.08)	0.051	0.79 (0.62–1.00)	0.970	1.01 (0.79–1.28)
rs4573233	GG	0.688	331 (66.73)	274 (63.57)	141 (61.84)	133 (65.52)				1 (Ref)		1 (Ref)
	GA		150 (30.24)	138 (32.02)	77 (33.77)	61 (30.05)	0.615	1.08 (0.80–1.47)	0.394	1.17 (0.82–1.67)	0.947	0.99 (0.68–1.44)
	AA		15 (3.03)	19 (4.41)	10 (4.39)	9 (4.93)	0.273	1.54 (0.71–3.32)	0.333	1.55 (0.64–3.76)	0.365	1.53 (0.61–3.80)
	AA+GA vs GG						0.442	1.12 (0.84–1.50)	0.297	1.20 (0.85–1.69)	0.850	1.04 (0.72–1.49)
	AA vs GG+GA						0.294	1.50 (0.71–3.18)	0.400	1.46 (0.61–3.48)	0.344	1.54 (0.63–3.78)
	A vs G						0.312	1.14 (0.89–1.46)	0.243	1.19 (0.89–1.60)	0.631	1.08 (0.79–1.47)
rs710885	TT	0.122	446 (88.32)	370 (85.65)	195 (85.53)	175 (85.78)				1 (Ref)		1 (Ref)
	TG		55 (10.89)	57 (13.19)	32 (14.04)	25 (12.25)	0.090	1.45 (0.94–2.21)	0.100	1.51 (0.92–2.48)	0.250	1.37 (0.80–2.32)
	GG		4 (0.79)	5 (1.16)	1 (0.43)	4 (1.97)	0.506	1.62 (0.39–6.76)	0.673	0.62 (0.06–5.88)	0.176	2.84 (0.63–12.93)
	GG+TG vs TT						0.072	1.46 (0.97–2.21)	0.133	1.45 (0.89–2.35)	0.132	1.48 (0.89–2.45)
	GG vs TT+TG						0.543	1.56 (0.37–6.54)	0.647	0.59 (0.06–5.65)	0.193	2.74 (0.60–12.51)
	G vs T						0.065	1.44 (0.98–2.11)	0.192	1.35 (0.86–2.14)	0.071	1.53 (0.96–2.44)
rs785005	GG	0.629	221 (44.92)	199 (45.96)	97 (42.73)	102 (49.51)				1 (Ref)		1 (Ref)
	GA		221 (44.92)	190 (43.88)	102 (44.94)	88 (42.72)	0.579	0.92 (0.68–1.24)	0.982	1.00 (0.70–1.43)	0.334	0.84 (0.58–1.20)
	AA		50 (10.16)	44 (10.16)	28 (12.33)	16 (7.77)	0.763	0.93 (0.57–1.52)	0.509	1.21 (0.69–2.11)	0.198	0.65 (0.34–1.25)
	AA+GA vs GG						0.570	0.92 (0.70–1.22)	0.808	1.04 (0.75–1.46)	0.216	0.80 (0.57–1.14)
	AA vs GG+GA						0.896	0.97 (0.61–1.54)	0.474	1.21 (0.71–2.04)	0.292	0.72 (0.39–1.33)
	A vs G						0.627	0.95 (0.77–1.17)	0.607	1.07 (0.83–1.37)	0.159	0.83 (0.64–1.08)
rs2632159	TT	0.991	357 (70.55)	281 (65.20)	142 (62.83)	139 (67.81)				1 (Ref)		1 (Ref)
	TC		136 (26.88)	130 (30.16)	77 (34.07)	53 (25.85)	0.129	1.27 (0.93–1.73)	**0.033**	**1.48 (1.03–2.12)**	0.789	1.06 (0.71–1.56)
	CC		13 (2.57)	20 (4.64)	7 (3.10)	13 (6.34)	**0.026**	**2.38 (1.11–5.09)**	0.304	1.67 (0.63–4.41)	**0.008**	**3.12 (1.34–7.23)**
	CC+TC vs TT						**0.040**	**1.37 (1.01–1.84)**	**0.022**	**1.51 (1.06–2.14)**	0.286	1.22 (0.85–1.77)
	CC vs TT+TC						**0.041**	**2.19 (1.03–4.64)**	0.445	1.46 (0.56–3.81)	**0.009**	**3.03 (1.32–6.94)**
	C vs T						**0.013**	**1.39 (1.07–1.79)**	**0.023**	**1.42 (1.05–1.92)**	0.061	1.35 (0.99–1.85)

Terms in bold represent significant results (*P*<0.05). *P*_HWE_ = *P* value for Hardy–Weinberg Equilibrium.Abbreviations: CRC, colorectal cancer; NA, not available. ^*^, the sort order was according to the SNP location in its genes from 5′ starting to 3′ ends. ^†^, *P* value was calculated by adjusted age and gender.

### Subgroup analysis

In the male subgroup, *PCAT1* rs2632159 SNP also increased rectal cancer risk by 3.97-fold in the male subgroup (*P*=0.017, Table 2), while *PCAT1* rs710885 polymorphism increased CRC risk by 2.09-fold and colon cancer risk by 2.60-fold (*P* = 0.033, *P* = 0.013, Table 2).

**Table 2:** Association of lnRNA-*PCAT1* polymorphisms with the CRC susceptibility stratified by host characteristics

SNP	Variable	Genotype	CON	CRC			CRC vs CON		Colon cancer vs CON		Rectal cancer vs CON	
				All	Colon cancer	Rectal cancer	P* value	OR (95% CI)	P* value	OR (95% CI)	P* value	OR (95% CI)
rs1902432	Gender											
	Male	TT	77	90	44	46		1 (Ref)		1 (Ref)		1 (Ref)
		TC	106	113	56	57	0.733	0.93 (0.59–1.45)	0.813	0.94 (0.55–1.59)	0.733	0.91 (0.54–1.54)
		CC	45	47	19	28	0.558	0.85 (0.48–1.49)	0.311	0.70 (0.35–1.40)	0.961	0.98 (0.52–1.87)
	Female	TT	93	70	46	24		1 (Ref)		1 (Ref)		1 (Ref)
		TC	131	95	55	40	0.973	0.99 (0.65–1.51)	0.608	0.88 (0.54–1.43)	0.531	1.20 (0.68–2.14)
		CC	47	19	8	11	0.109	0.59 (0.31–1.13)	0.028	0.38 (0.16–0.90)	0.908	0.95 (0.43–2.14)
	Age											
	≦60	TT	120	80	40	40		1 (Ref)		1 (Ref)		1 (Ref)
		TC	173	106	59	47	0.679	0.92 (0.64–1.34)	0.925	1.02 (0.64–1.63)	0.436	0.83 (0.51–1.34)
		CC	75	32	14	18	0.073	0.63 (0.38–1.04)	0.088	0.56 (0.28–1.09)	0.279	0.71 (0.38–1.33)
	>60	TT	50	80	50	30		1 (Ref)		1 (Ref)		1 (Ref)
		TC	64	102	52	50	0.830	0.95 (0.58–1.54)	0.399	0.79 (0.46–1.36)	0.495	1.24 (0.67–2.26)
		CC	17	34	13	21	0.867	1.06 (0.52–2.15)	0.388	0.69 (0.30–1.60)	0.206	1.70 (0.75–3.84)
rs4573233	Gender											
	Male	GG	152	160	77	83		1 (Ref)		1 (Ref)		1 (Ref)
		GA	66	79	37	42	0.815	1.06 (0.67–1.65)	0.933	1.02 (0.60–1.75)	0.759	1.08 (0.65–1.82)
		AA	5	11	5	6	0.090	2.90 (0.85–9.93)	0.162	2.69 (0.67–10.73)	0.102	3.14 (0.80–12.33)
	Female	GG	10	8	64	50		1 (Ref)		1 (Ref)		1 (Ref)
		GA	84	59	40	19	0.659	1.10 (0.72–1.67)	0.248	1.33 (0.82–2.16)	0.485	0.81 (0.45–1.47)
		AA	179	114	5	3	0.890	1.07 (0.39–2.96)	0.818	1.15 (0.35–3.72)	0.961	0.97 (0.25–3.75)
	Age											
	≦60	GG	254	142	75	67		1 (Ref)		1 (Ref)		1 (Ref)
		GA	104	67	35	32	0.450	1.15 (0.80–1.67)	0.579	1.14 (0.72–1.81)	0.525	1.17 (0.72–1.89)
		AA	10	7	3	4	0.659	1.25 (0.47–3.35)	0.985	1.01 (0.27–3.78)	0.495	1.51 (0.46–4.98)
	>60	GG	77	132	66	66		1 (Ref)		1 (Ref)		1 (Ref)
		GA	46	71	42	29	0.749	0.92 (0.57–1.50)	0.792	1.08 (0.63–1.85)	0.343	0.75 (0.41–1.36)
		AA	5	12	7	5	0.352	1.71 (0.55–5.28)	0.315	1.87 (0.55–6.32)	0.561	1.50 (0.39–5.81)
rs710885	Gender											
	Male	TT	210	217	102	115		1 (Ref)		1 (Ref)		1 (Ref)
		TG	19	31	18	13	**0.033**	**2.09 (1.06–4.11)**	**0.013**	**2.60 (1.22–5.55)**	0.222	1.65 (0.74–3.68)
		GG	1	3	0	3	0.506	2.20 (0.22–22.60)	NA	NA	0.233	4.12 (0.40–42.27)
	Female	TT	236	153	93	60		1 (Ref)		1 (Ref)		1 (Ref)
		TG	36	26	14	12	0.657	1.14 (0.65–1.99)	0.985	1.01 (0.51–.99)	0.438	1.33 (0.65–2.74)
		GG	3	2	1	1	0.862	1.18 (0.18–7.62)	0.986	0.98 (0.10–10.10)	0.741	1.48 (0.15–15.10)
	Age											
	≦60	TT	327	181	95	86		1 (Ref)		1 (Ref)		1 (Ref)
		TG	40	33	18	15	0.106	1.51 (0.92–2.47)	0.152	1.55 (0.85–2.84)	0.254	1.45 (0.77–2.76)
		GG	4	1	0	1	0.491	0.46 (0.05–4.17)	NA	NA	0.997	1.01 (0.11–9.20)
	>60	TT	119	189	100	89		1 (Ref)		1 (Ref)		1 (Ref)
		TG	15	24	14	10	0.677	1.16 (0.57–2.37)	0.607	1.23 (0.56–2.71)	0.878	1.07 (0.44–2.60)
		GG	0	4	1	3	NA	NA	NA	NA	NA	NA
rs785005	Gender											
	Male	GG	99	121	53	68		1 (Ref)		1 (Ref)		1 (Ref)
		GA	96	107	54	53	0.528	0.87 (0.56–1.35)	0.997	1.00 (0.60–1.68)	0.298	0.77 (0.46–1.27)
		AA	28	24	13	11	0.395	0.73 (0.35–1.51)	0.824	0.91 (0.39–2.12)	0.240	0.60 (0.26–1.41)
	Female	GG	122	78	44	34		1 (Ref)		1 (Ref)		1 (Ref)
		GA	125	83	48	35	0.936	0.98 (0.65–1.48)	0.989	1.00 (0.61–1.64)	0.876	0.96 (0.56–1.65)
		AA	22	20	15	5	0.458	1.30 (0.65–2.59)	0.157	1.73 (0.81–3.70)	0.576	0.74 (0.26–2.14)
	Age											
	≦60	GG	169	96	48	48		1 (Ref)		1 (Ref)		1 (Ref)
		GA	160	98	51	47	0.655	1.09 (0.76–1.55)	0.607	1.13 (0.72–1.76)	0.855	1.04 (0.66–1.65)
		AA	52	103	14	10	0.709	1.12 (0.63–1.97)	0.413	1.34 (0.67–2.68)	0.794	0.90 (0.42–1.95)
	>60	GG	52	103	49	54		1 (Ref)		1 (Ref)		1 (Ref)
		GA	61	92	51	41	0.320	0.78 (0.48–1.27)	0.706	0.90 (0.52–1.56)	0.160	0.66 (0.37–1.18)
		AA	12	20	14	6	0.880	094 (0.40–2.20)	0.552	1.32 (0.53–3.27)	0.259	0.53 (0.17–1.61)
rs2632159	Gender											
	Male	TT	169	166	75	91		1 (Ref)				
		TC	56	71	41	30	0.396	1.22 (0.77–1.94)	0.100	1.56 (0.92–2.65)	0.829	0.94 (0.54–1.64)
		CC	6	12	2	10	0.081	2.64 (0.89–7.82)	0.978	0.98 (0.18–5.23)	**0.017**	**3.97 (1.28–12.25)**
	Female	TT	188	115	67	48		1 (Ref)				
		TC	80	59	36	23	0.269	1.27 (0.83–1.93)	0.250	1.34 (0.82–2.20)	0.580	1.17 (0.67–2.07)
		CC	7	8	5	3	0.164	2.14 (0.73–6.23)	0.167	2.35 (0.70–7.92)	0.385	1.86 (0.46–7.61)
	Age											
	≦60	TT	262	140	71	69		1 (Ref)		1 (Ref)		1 (Ref)
		TC	97	65	37	28	0.185	1.73 (0.77–3.90)	0.139	1.42 (0.89–2.25)	0.679	1.11 (0.68–1.83)
		CC	13	12	5	7	0.218	1.27 (0.87–1.84)	0.522	1.42 (0.49–4.11)	0.140	2.06 (0.79–5.39)
	>60	TT	95	141	71	70		1 (Ref)		1 (Ref)		1 (Ref)
		TC	39	65	40	25	NA	NA	0.240	1.39 (0.80–2.40)	0.706	0.89 (0.48–1.65)
		CC	0	8	2	6	0.563	1.16 (0.71–1.89)	NA	NA	NA	NA

Terms in bold represent significant results (*P*<0.05).

^*^using Logistic Regression adjusted by the two factors of gender and age. Abbreviations: CON, controls; CRC, colorectal cancer. NA, not available.

### Haplotype analysis

In the haplotype analysis, no haplotype of the *PCAT1* gene had any association with CRC risk (haplotype for rs1902432-rs4573233-rs710885-rs785005-rs2632159 SNPs, *P*>0.05). However, when cases were divided into colon cancer and rectal cancer, the *PCAT1* gene C-G-T-G-T haplotype decreased colon cancer risk to 0.75-fold (*P*=0.019, Table 3).

**Table 3:** The association of haplotype of *PCAT1* gene and CRC risk

Haplotype	Control (%)	CRC			CRC vs CON		Colon cancer vs CON		Rectal cancer vs CON	
		All (%)	Colon cancer (%)	Rectal cancer (%)	*P* value	OR (95% CI)	*P* value	OR (95% CI)	*P* value	OR (95% CI)
C G T G T	395.24 (41.6)	326.83 (39.0)	154.44 (34.9)	171.99 (43.4)	0.238	0.89 (0.74–1.08)	**0.019**	**0.75 (0.60–0.95)**	0.615	1.06 (0.84–1.35)
T A T G C	85.64 (9.0)	91.75 (10.9)	50.77 (11.5)	41.79 (10.6)	0.178	1.24 (0.91–1.69)	0.145	1.31 (0.91–1.90)	0.405	1.18 (0.80–1.74)
T A T G T	88.36 (9.3)	76.25 (9.1)	41.23 (9.3)	34.21 (8.6)	0.866	0.97 (0.71–1.34)	0.979	1.01 (0.68–1.48)	0.669	0.91 (0.60–1.38)
T G G G C	56.52 (5.9)	64.70 (7.7)	31.64 (7.2)	33.00 (8.3)	0.142	1.32 (0.91–1.91)	0.384	1.22 (0.78–1.92)	0.119	1.43 (0.91–2.23)
T G T A T	308.92 (32.5)	267.57 (31.9)	155.98 (35.3)	111.85 (28.2)	0.755	0.97 (0.79–1.18)	0.294	1.14 (0.90–1.44)	0.102	0.81 (0.62–1.04)

The bold terms means the significant results (*P*<0.05). Using SHEsis software to analysis (http://analysis.bio-x.cn/). The haplotype for rs1902432 – rs4573233 -rs710885 – rs785005 – rs2632159 SNPs. CRC: colorectal cancer.

### Epistatic effect analysis

To mine the associations of the SNPs with positive results, rs2632159, and other SNPs with the risk of CRC, the epistatic effects of rs2632159 interacting with four other SNPs were calculated. When there is rs2632159 polymorphism, the rs1902432 TT+TC variant genotype was shown to increase rectal cancer risk 2.93-fold (*P*=0.011, 95% CI = 1.28–6.70), and the rs785005 GG variant genotype increased colon cancer risk 2.01-fold (*P*=0.008, 95% CI = 1.21–3.28, Table 4).

**Table 4:** Epistatic effect of pairwise interacting factors for *PCAT1* on the risks of CRC

Interacted pairwise SNPs	Comparison	Subset	CRC vs CON		Colon cancer vs CON		Rectal cancer vs CON	
			*P* value	OR (95% CI)	*P* value	OR (95% CI)	*P* value	OR (95% CI)
rs1902432 interacted with rs2632159	rs1902432 CC vs TT+TC	rs2632159 CC	NA	NA	NA	NA	NA	NA
		rs2632159 TT+TC	0.196	0.78 (0.54–1.14)	0.025	0.57 (0.35–0.93)	0.871	1.04 (0.67–1.62)
	rs2632159 CC vs TT+TC	rs1902432 CC	NA	NA	NA	NA	NA	NA
		rs1902432 TT+TC	0.065	2.02 (0.96–4.27)	0.603	1.29 (0.49–3.37)	**0.011**	**2.93 (1.28–6.70)**
rs4573233 interacted with rs2632159	rs4573233 AA+GA vs GG	rs2632159 CC	0.547	0.85 (0.51–1.44)	NA	NA	NA	NA
		rs2632159 TT+TC	0.788	1.06 (0.70–1.61)	0.441	1.15 (0.81–1.64)	0.820	0.96 (0.67–1.40)
	rs2632159 CC vs TT+TC	rs4573233 AA+GA	0.406	1.23 (0.76–1.98)	0.200	1.45 (0.82–2.57)	0.991	1.00 (0.55–1.83)
		rs4573233 GG	0.063	1.55 (0.98–2.45)	0.101	1.57 (0.92–2.69)	0.141	1.52 (0.87–2.66)
rs710885 interacted with rs2632159	rs710885 GG+TG vs TT	rs2632159 CC	0.266	1.34 (0.80–2.23)	0.310	0.16 (0.01–5.49)	0.195	0.11 (0.00–3.09)
		rs2632159 TT+TC	0.570	0.58 (0.09–3.77)	0.164	1.45 (0.86–2.44)	0.310	1.34 (0.76–2.34)
	rs2632159 CC vs TT+TC	rs710885 GG+TG	0.189	3.84 (0.52–28.60)	0.516	1.95 (0.26–14.50)	NA	NA
		rs710885 TT	0.321	1.20 (0.84–1.72)	0.109	1.40 (0.93–2.13)	0.959	0.99 (0.62–1.56)
rs785005 interacted with rs2632159	rs785005 AA+GA vs GG	rs2632159 CC	0.672	0.89 (0.53–1.51)	NA	NA	NA	NA
		rs2632159 TT+TC	0.643	1.09 (0.76–1.55)	0.496	1.13 (0.80–1.59)	0.425	0.87 (0.61–1.23)
	rs2632159 CC vs TT+TC	rs785005 AA+GA	0.390	1.22 (0.77–1.94)	0.388	1.27 (0.74–2.16)	0.590	1.17 (0.66–2.10)
		rs785005 GG	0.051	1.55 (1.00–2.40)	**0.008**	**2.01 (1.21–3.28)**	0.540	1.18 (0.70–1.98)

Terms in bold represent significant results (*P*<0.05). All tests were adjusted by age and gender. Statistically significant associations were highlighted in bold (*P*<0.05). Abbreviations: CON, controls; CRC, colorectal cancer.

### Association of SNPs and clinical pathological parameters

We mined the distribution frequencies of five SNPs in association with different clinical pathological parameters and found in all CRC patients that the frequency of genotypes with the rs785005 SNP variant was higher in the cases with high/moderate-differentiation adenocarcinoma (variant genotype vs heterozygote/wild-type genotype: 97.5% vs 83.0%/81.5%, Table 5). Meanwhile, in colon cancer patients, the frequency of rs4573233 SNP variant genotype was higher in poor-differentiation adenocarcinoma (variant genotype vs heterozygote/wild-type genotype: 62.5% vs 83.3%/86.2%, Table 5). Moreover, the frequency of the rs2632159 SNP variant genotype was higher in the never smokers (variant genotype vs heterozygote/wild-type genotype: 100.0% vs 61.0%/64.1%, Table 5).

**Table 5:** The association between the genotype of *PCAT1* SNPs and clinicopathological parameters^*^

Parameter	*n*	Genotype for CRC			*P* value	Genotype for Colon cancer			*P* value	Genotype for Rectal cancer			*P* value
		Wild-type (%)	Heterozygote (%)	Variant type (%)		Wild-type (%)	Heterozygote (%)	Variant type (%)		Wild-type (%)	Heterozygote (%)	Variant type (%)	
rs1902432													
Smoking					0.068				0.130				0.392
Ever smoker	173 (39.86)	64 (40.0)	76 (36.5)	33 (50.0)		30 (33.3)	37 (33.3)	13 (48.1)		34 (48.6)	39 (40.2)	20 (51.3)	
Never smoker	261 (60.14)	96 (60.0)	132 (63.5)	33 (50.0)		60 (66.7)	74 (66.7)	14 (51.9)		36 (51.4)	58 (59.8)	19 (48.7)	
Drinking					0.765				0.439				0.763
Drinker	48 (11.06)	20 (12.5)	20 (9.6)	8 (12.1)		9 (10.0)	11 (9.9)	4 (14.8)		11 (15.7)	9 (9.3)	4 (10.3)	
Nondrinker	386 (88.94)	140 (87.5)	188 (90.4)	58 (87.9)		81 (90.0)	100 (90.1)	23 (85.2)		59 (84.3)	88 (90.7)	35 (89.7)	
Family history					0.207				0.772				0.097
Yes	25 (5.76)	8 (5.0)	11 (5.3)	6 (9.1)		6 (6.7)	4 (3.6)	1 (3.7)		2 (2.9)	7 (7.2)	5 (12.8)	
No	409 (94.24)	152 (95.0)	197 (94.7)	60 (90.9)		84 (93.3)	107 (96.4)	26 (96.3)		68 (97.1)	90 (92.8)	34 (87.2)	
WHO classification					0.603				0.052				0.664
High-moderate differentiation	322 (83.64)	123 (86.6)	149 (82.3)	50 (80.6)		71 (87.7)	81 (83.5)	20 (76.9)		52 (85.2)	68 (81.0)	30 (83.3)	
Poor differentiation	21 (5.45)	6 (4.2)	10 (5.5)	5 (8.1)		2 (2.5)	5 (5.2)	4 (15.4)		4 (6.6)	5 (6.0)	1 (2.8)	
Mucinous adenocarcinoma	42 (10.91)	13 (9.2)	22 (12.2)	7 (11.3)		8 (9.9)	11 (11.3)	2 (7.7)		5 (8.2)	11 (13.1)	5 (13.9)	
TNM stage					0.506				0.185				0.690
I–II	174 (40.65)	64 (40.0)	86 (42.4)	24 (36.9)		35 (38.9)	53 (49.1)	8 (30.8)		29 (41.4)	33 (34.7)	16 (41.0)	
III–IV	254 (59.35)	96 (60.0)	117 (57.8)	41 (63.1)		55 (61.1)	55 (50.9)	18 (69.2)		41 (58.6)	62 (65.3)	23 (59.0)	
rs4573233													
Smoking					0.858				0.730				0.548
Ever smoker	173 (40.14)	108 (39.4)	57 (41.3)	8 (42.1)		51 (36.2)	26 (33.8)	3 (30.0)		57 (42.9)	31 (50.8)	5 (55.6)	
Never smoker	258 (59.86)	166 (60.6)	81 (58.7)	11 (57.9)		90 (63.8)	51 (66.2)	7 (70.0)		76 (57.1)	30 (49.2)	4 (44.4)	
Drinking					0.931				0.267				0.323
Drinker	48 (11.14)	29 (10.6)	17 (12.3)	2 (10.5)		16 (11.3)	8 (10.4)	0		13 (9.8)	9 (14.8)	2 (22.2)	
Nondrinker	383 (88.86)	245 (89.4)	121 (87.7)	17 (89.5)		125 (88.7)	69 (89.6)	10 (100.0)		120 (90.2)	52 (85.2)	7 (77.8)	
Family history					0.918				0.435				0.404
Yes	25 (5.80)	21 (7.7)	3 (2.2)	1 (5.3)		9 (6.4)	1 (1.3)	1 (10.0)		12 (9.0)	2 (3.3)	0	
No	406 (94.20)	253 (92.3)	135 (97.8)	18 (94.7)		132 (93.6)	76 (98.7)	9 (90.0)		121 (91.0)	59 (96.7)	9 (100.0)	
WHO classification					0.055				**0.040**				0.435
High-moderate differentiation	320 (83.77)	214 (85.3)	94 (81.7)	12 (75.0)		112 (86.2)	55 (83.3)	5 (62.5)		102 (84.3)	39 (79.6)	7 (87.5)	
Poor differentiation	21 (5.50)	11 (4.4)	7 (6.1)	3 (18.8)		6 (4.6)	3 (4.5)	2 (25.0)		5 (4.1)	4 (8.2)	1 (12.5)	
Mucinous adenocarcinoma	41 (10.73)	26 (10.4)	14 (12.2)	1 (6.3)		12 (9.2)	8 (12.1)	1 (12.5)		14 (11.6)	6 (12.2)	0	
TNM stage					0.560				0.852				0.291
I–II	174 (40.94)	109 (40.2)	56 (41.5)	9 (47.4)		57 (41.3)	35 (46.1)	4 (40.0)		52 (39.1)	21 (35.6)	5 (55.6)	
III–IV	251 (59.06)	162 (59.8)	79 (58.5)	10 (52.6)		81 (58.7)	41 (53.9)	6 (60.0)		81 (60.9)	38 (64.4)	4 (44.4)	
													
rs710885													
Smoking					0.352				0.457				0.404
Ever smoker	174 (40.28)	148 (40.0)	25 (43.9)	1 (20.0)		68 (34.9)	13 (40.6)	0		80 (45.7)	12 (48.0)	1 (25.0)	
Never smoker	258 (59.72)	222 (60.0)	32 (56.1)	4 (80.0)		127 (65.1)	19 (59.4)	1 (100.0)		95 (54.3)	13 (52.0)	3 (75.0)	
Drinking					0.510				0.731				0.381
Drinker	47 (10.88)	41 (11.1)	5 (8.8)	1 (20.0)		21 (10.8)	3 (9.4)	0		20 (11.4)	2 (8.0)	1 (25.0)	
Nondrinker	385 (89.12)	329 (88.9)	52 (91.2)	4 (80.0)		174 (89.2)	29 (90.6)	1 (100.0)		155 (88.6)	23 (92.0)	3 (75.0)	
Family history					0.156				0.830				0.147
Yes	24 (5.56)	21 (5.7)	2 (3.5)	1 (20.0)		9 (4.6)	1 (3.1)	0		12 (6.9)	1 (4.0)	1 (25.0)	
No	408 (94.44)	349 (94.3)	55 (96.5)	4 (80.0)		186 (95.4)	31 (96.9)	1 (100.0)		163 (93.1)	24 (96.0)	3 (75.0)	
WHO classification					0.682				0.914				0.736
High-moderate differentiation	322 (84.07)	272 (83.4)	46 (86.8)	4 (100.0)		147 (85.0)	25 (83.3)	1 (100.0)		125 (81.7)	21 (91.3)	3 (100.0)	
Poor differentiation	20 (5.22)	18 (5.5)	2 (3.8)	0		10 (5.8)	1 (3.3)	0		8 (5.2)	1 (4.3)	0	
Mucinous adenocarcinoma	41 (10.71)	36 (11.0)	5 (9.4)	0		16 (9.2)	4 (13.3)	0		20 (13.1)	1 (4.3)	0	
TNM stage					0.961				0.381				0.637
I–II	175 (41.08)	147 (40.4)	26 (45.6)	2 (40.0)		83 (43.5)	14 (43.8)	0		64 (37.0)	12 (48.0)	2 (50.0)	
III–IV	251 (58.92)	217 (59.6)	31 (54.4)	3 (60.0)		108 (56.5)	18 (56.3)	1 (100.0)		109 (63.0)	13 (52.0)	2 (50.0)	
rs785005													
Smoking					0.121				0.208				0.497
Ever smoker	175 (40.42)	84 (42.2)	78 (41.1)	13 (29.5)		35 (36.1)	39 (38.2)	7 (25.0)		49 (48.0)	39 (44.3)	6 (37.5)	
Never smoker	258 (59.58)	115 (57.8)	112 (58.9)	31 (70.5)		62 (63.9)	63 (61.8)	21 (75.0)		53 (52.0)	49 (55.7)	10 (62.5)	
Drinking					0.657				0.979				0.483
Drinker	385 (88.91)	21 (10.6)	23 (12.1)	4 (9.1)		9 (9.3)	12 (11.8)	3 (10.7)		12 (11.8)	11 (12.5)	1 (6.3)	
Nondrinker	48 (11.09)	178 (89.4)	167 (87.9)	40 (90.9)		88 (90.7)	90 (88.2)	25 (89.3)		90 (88.2)	77 (87.5)	15 (93.7)	
Family history					0.320				0.122				0.928
Yes	25 (5.77)	12 (6.0)	9 (4.7)	4 (9.1)		3 (3.1)	5 (4.9)	3 (10.7)		9 (8.8)	4 (4.5)	1 (6.3)	
No	408 (94.23)	187 (94.0)	181 (95.3)	40 (90.9)		94 (94.0)	97 (95.1)	25 (89.3)		93 (91.2)	84 (95.5)	15 (93.8)	
WHO classification					**0.045**				0.068				0.527
High-moderate differentiation	322 (83.85)	141 (81.5)	142 (83.0)	39 (97.5)		67 (78.8)	79 (85.9)	26 (100.0)		74 (84.1)	63 (79.7)	13 (92.9)	
Poor differentiation	20 (5.21)	13 (7.5)	7 (4.1)	0		9 (10.6)	1 (1.1)	0		4 (4.5)	6 (7.6)	0	
Mucinous adenocarcinoma	42 (10.94)	19 (11.0)	22 (12.9)	1 (2.5)		9 (10.6)	12 (13.0)	0		10 (11.4)	10 (12.7)	1 (7.1)	
TNM stage					0.992				0.631				0.636
I–II	175 (40.98)	82 (42.3)	75 (39.7)	18 (40.9)		41 (43.6)	45 (44.6)	11 (39.3)		41 (41.0)	30 (34.1)	7 (43.8)	
III–IV	252 (59.02)	112 (57.7)	114 (60.3)	26 (59.1)		53 (56.4)	56 (55.4)	17 (60.7)		59 (59.0)	58 (65.9)	9 (56.3)	
rs2632159													
Smoking					0.616				**0.045**				0.526
Ever smoker	174 (40.37)	115 (40.9)	52 (40.0)	7 (35.0)		51 (35.9)	30 (39.0)	0		64 (46.0)	22 (41.5)	7 (53.8)	
Never smoker	257 (59.63)	166 (59.1)	78 (60.0)	13 (65.0)		91 (64.1)	47 (61.0)	7 (100.0)		75 (54.0)	31 (58.5)	6 (46.2)	
Drinking					0.869				0.354				0.670
Drinker	48 (11.14)	31 (11.0)	15 (11.5)	2 (10.0)		16 (11.3)	8 (10.4)	0		15 (10.8)	7 (13.2)	2 (15.4)	
Nondrinker	383 (88.86)	250 (89.0)	115 (88.5)	18 (90.0)		126 (88.7)	69 (89.6)	7 (100.0)		124 (89.2)	46 (86.8)	11 (84.6)	
Family history									0.543				0.899
Yes	25 (5.80)	19 (6.8)	5 (3.8)	1 (5.0)	0.875	7 (4.9)	4 (5.2)	0		12 (8.6)	1 (1.9)	1 (7.7)	
No	406 (94.20)	262 (93.2)	125 (96.2)	19 (95.0)		135 (95.1)	73 (94.8)	7 (100.0)		127 (91.4)	52 (98.1)	12 (92.3)	
WHO classification					0.991				0.158				0.422
High-moderate differentiation	319 (83.51)	215 (84.3)	90 (81.8)	14 (82.4)		111 (86.0)	55 (82.1)	4 (66.7)		104 (82.5)	35 (81.4)	10 (90.9)	
Poor differentiation	21 (5.50)	15 (5.9)	5 (4.5)	1 (5.9)		8 (6.2)	3 (4.5)	0		7 (5.6)	2 (4.7)	1 (9.1)	
Mucinous adenocarcinoma	42 (10.99)	25 (9.8)	15 (13.6)	2 (11.8)		10 (7.8)	9 (13.4)	2 (33.3)		15 (11.9)	6 (14.0)	0	
TNM stage					0.183				0.436				0.237
I–II	173 (40.71)	106 (38.0)	56 (44.4)	11 (55.0)		56 (40.0)	35 (46.7)	4 (57.1)		50 (36.0)	21 (41.2)	7 (53.8)	
III–IV	252 (59.29)	173 (62.0)	70 (55.6)	9 (45.0)		84 (60.0)	40 (53.3)	3 (42.9)		89 (64.0)	30 (58.8)	6 (46.2)	

Terms in bold represent significant results (*P*<0.05). The *P* value was calculated using the recessive model.^*^, using χ^2^ two-side test.

### eQTL analysis

**Figure 1:**
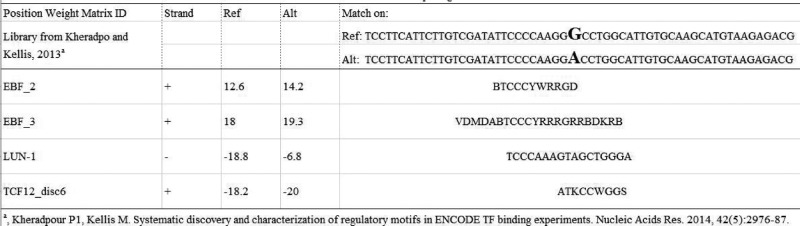
The bioinformatical analysis for rs2632159 SNP In the HaploReg database, the promising SNP rs2632159 was found possibly to change the combination site with the following proteins with the certain polypeptide.

In the HaploReg database, the promising SNP rs2632159 was found to potentially change the site of binding with EBF, LUN-1, and TCF12, which are transcription factors [18] (Figure 1).

## Discussion

To date, no study has reported on the association of *PCAT1* polymorphisms and CRC risk. We thus screened all tagSNPs for the *PCAT1* gene and investigated whether these SNPs could be biomarkers for CRC risk. We reported for the first time that *PCAT1* rs2632159 polymorphism could increase the risk of CRC. We then performed an eQTL analysis and found that rs2632159 polymorphism could alter the site of binding with three different transcription factors, which may explain why rs2632159 polymorphism increased CRC risk. Our study should be helpful in the search for promising biomarkers for the prediction of CRC at an early stage.

These five tagSNPs represented all of the SNPs covering the *PCAT1* gene. In terms of the locations, rs1902432 and rs4573233 polymorphisms are intronic and in the region 2 kb upstream of the gene, respectively; rs710885 and rs785005 are intronic; and rs2632159 is 145 bp downstream of the gene. Thus far, no study has reported the association of *PCAT1* polymorphisms and CRC risk. Only the *PCAT1* rs1902432 SNP was reported to be significantly associated with other cancer, namely, increasing the risk of prostate cancer [7]. In our study, we found that the rs1902432 SNP was associated with colon cancer risk, but not with disease risk in all CRC patients. Thus, rs1902432 SNP may only have potential to be a biomarker for colon cancer risk.

The major finding in the present study was that the *PCAT1* rs2632159 SNP increased CRC susceptibility, and functioned as a risk factor for colon cancer under the dominant model and for rectal cancer under the recessive model. This SNP was thus reported to be related to disease risk for the first time. The explanation for this phenomenon should be considered from two aspects: first, the etiology of colon cancer and rectal cancer is different. The etiology of colon cancer is often related to genetic factors. Its precancerous disease may be familial polyposis, and its patients often have microsatellite instability, for example, MLH1, MSH2, MSH6 and PMS2 deficiency. While the etiology of rectal cancer is often related to the unhealthy dietary habit, and patients often have poor defecation habits, resulting in a large number of toxins accumulated in the rectum, resulting in repeated irritation of rectal mucosa by inflammation, thus resulting the occurrence of colon cancer. Second, the basis of genetics varies from the dominant model and recessive model. The dominant model is a single variation for the allele of one polymorphism, while the recessive model needs to achieve double variation for two alleles of one polymorphism. This is similar to the ‘two-hit hypothesis’ [19] in genetics.

*PCAT1* rs2632159 SNP was also found to have a more pronounced effect in the male subgroup. This SNP was also shown to have epistatic effects with other polymorphisms. Epistasis is a phenomenon in which the effects of complex interactions are more important than the independent main effects of any one susceptibility gene [20–22]. When rs2632159 polymorphism is present, the rs1902432 TT+TC variant genotype was shown to increase the risk of rectal cancer, and the rs785005 GG variant genotype increased the risk of colon cancer; this suggests the potential for pairwise effects of SNP–SNP interaction in the *PCAT1* gene. Finally, eQTL analysis using an online bioinformatic tool was performed for this promising rs2632159 polymorphism, and EBF, LUN-1, and TCF12 were found to probably combine with the rs2632159 T>C variant. Then, this rs2632159 T>C variant would cause a gain or loss function for these transcription factors. PCAT1 is a lncRNA that functions as an oncogene [23–28]. We thus speculate that these three transcription factors could stimulate PCAT1 transcription and increase the level of oncogene functional PCAT1 expression, causing individuals carrying the variant genotype to express more oncogenic PCAT1 and therefore increase the risk of CRC.

**Figure 2:**
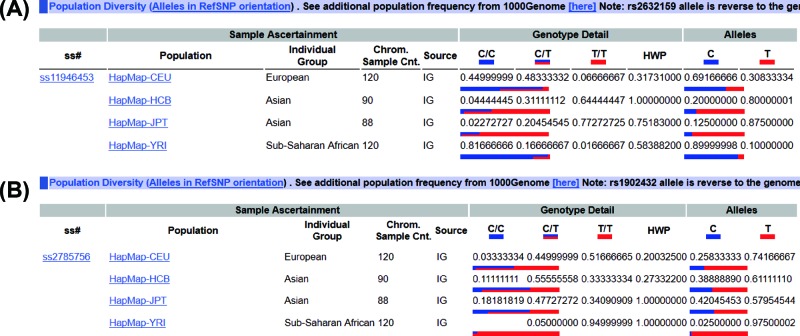
The genotype information from Hapmap database The genotype for *PCAT1* rs2632159 and rs1902432 SNPs from the Hapmap database (https://www.ncbi.nlm.nih.gov/projects/SNP/snp_ref.cgi?do_not_redirect&rs=rs2632263; https://www.ncbi.nlm.nih.gov/projects/SNP/snp_ref.cgi?do_not_redirect&rs=rs1902432.)

Gene research may be related to race and this will limit some application of the SNP for the different ethnicity. For example, our research found that *PCAT1* rs2632159 SNP was shown to increase CRC susceptibility in a Chinese population, and the rs1902432 SNP might only have potential to be a biomarker for colon cancer risk in the Chinese population. While the *PCAT1* rs1902432 SNP was also reported to be significantly associated with the risk of prostate cancer in Chinese population [7]. In Figure 2, we listed the Hapmap data for the *PCAT1* rs2632159 and rs1902432 SNPs, which showed rs2632159 varied from Europeans to Asians to Africans (the T allele percentage, 30% in Europeans, 80% in Asians, 10% in Africans), so was the rs1902432 SNP. Thus, the application of *PCAT1* rs2632159 and rs1902432 SNPs for cancer risk might be considered from race to race.

It was reported that the combined haplotype analyses for multiple SNPs are more sensitive and powerful than single-locus SNP analysis alone [29]. The single-locus SNP rs4573233, rs710885 and rs785005 SNPs alone had no significant association with colon risk, but when these three SNPs composed a combination with others (rs1902432 and rs2632159), we found that the *PCAT1* C G T G T haplotype of rs1902432 – rs4573233 -rs710885 – rs785005 – rs2632159 SNPs significantly decreased the colon risk (OR = 0.75), indicating that the haplotype might be more sensitive than these three single SNP its own, and could also have potential for the prediction of colon cancer risk.

There are some limitations in the present study. First, the sample size could be increased to obtain more reliable results, but the total sample size of nearly 1000 is reasonable. Second, the prognostic data could have been studied to achieve a more comprehensive analysis, but the survival rate of CRC was high, which limited the predictive role of the SNP for CRC prognosis. Third, whether the *PCAT1* rs2632159 associated with other gastrointestinal cancers were not studied in this research, which would determine the application and specificity of the *PCAT1* polymorphisms.

In summary, in the present study, the *PCAT1* rs2632159 SNP was shown to increase CRC susceptibility, and it was shown to increase the risk of colon cancer under the dominant model and of rectal cancer under the recessive model. In the subgroup analysis, the *PCAT1* rs2632159 SNP also increased the risk of rectal cancer in males. And the rs1902432 SNP might only have potential to be a biomarker for colon cancer risk. These findings should help in the search for biomarkers predictive of CRC susceptibility and provide more clues to obtain a deeper understanding of the etiology of colorectal carcinogenesis.

## Supporting information

**Supplementary Figure S1:** 

**Supplementary Table S1:** The captured SNPs covered by the selected tagSNPs

**Supplementary Table S2:** The baseline of the subjects

## Abbreviations

CI: confidence interval
CON: controls
CRC: colorectal cancer
eQTL: quantitative trait locus
LD: linkage disequilibrium
OR: odds ratio
PCAT: prostate cancer-associated transcript
SNP: single nucleotide polymorphism
