# Supplementary material for: lncRNA-PCAT1 rs2632159 polymorphism could be a biomarker for colorectal cancer susceptibility
Source: Biosci Rep. 2019 Jul 12;39(7):BSR20190708. doi: 10.1042/BSR20190708 (PMC6629943; doi:10.1042/BSR20190708)
Supplement: Supplementary file 1 [file bsr20190708_Supp1.pdf]

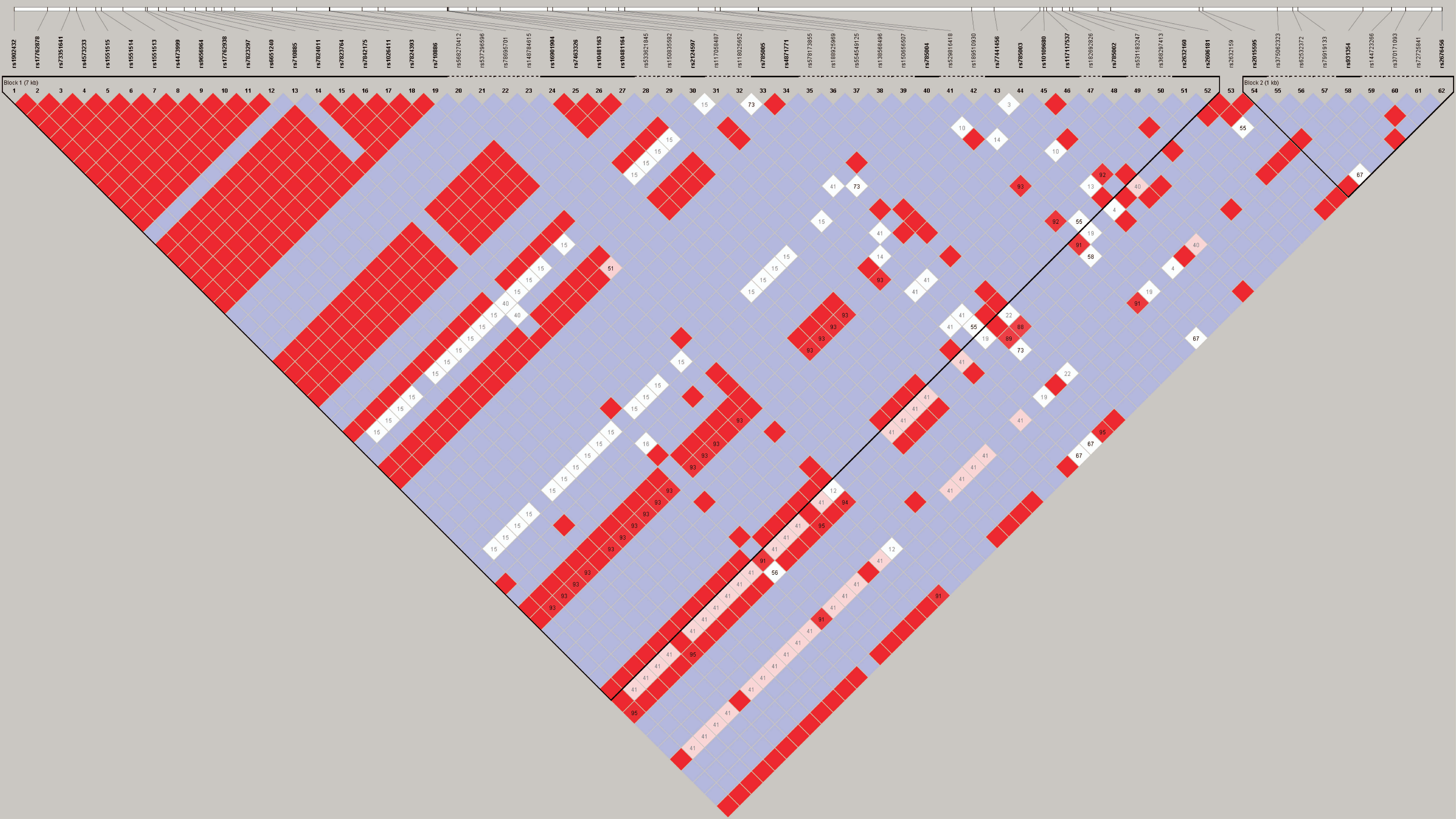
rs1902432
rs17762878
rs73351641
rs4573233
rs1551515
rs1551514
rs1551513
rs4473999
rs9656964
rs17762938
rs7823297
rs6651240
rs710885
rs7824011
rs7823764
rs7842175
rs1026411
rs7824393
rs710886
rs568270412
rs537296596
rs78695701
rs148784615
rs16901904
rs7463326
rs10481163
rs10481164
rs533621845
rs150835582
rs2124597
rs117058487
rs118025652
rs785005
rs4871771
rs578173855
rs188925969
rs554549125
rs138568496
rs150656507
rs785004
rs529816418
rs189510930
rs77441456
rs785003
rs10109680
rs117117537
rs182692826
rs785002
rs531183247
rs368297413
rs2632160
rs2606181
rs2632159
rs2015595
rs375062323
rs62532372
rs79919133
rs931354
rs144723266
rs370171093
rs72725841
rs2676456
Block 1 (7 kb)
Block 2 (1 kb)

<Supplementary Materials>

lncRNA-*PCAT1* rs2632159 polymorphism could be a biomarker for colorectal cancer susceptibility

Short title: *PCAT1* SNP for CRC risk

Ming-li Yang[1], Zhe Huang[2], Li-na Wu[1], Rong Wu[1], Han-xi Ding[3], Ben-gang Wang[4,*]

[1] The 2nd Oncology Department of Affiliated Shengjing Hospital of China Medical University, Shenyang 110022, China

[2]Genery Surgery Department of Affiliated Shengjing Hospital of China Medical University, Shenyang 110015, China

[3]Tumor Etiology and Screening Department of Cancer Institute and General Surgery, the First Affiliated Hospital of China Medical University, Shenyang 110001, China

[4]Department 1 of General Surgery, the First Hospital of China Medical University, Shenyang 110001, China

***Corresponding author:** Dr. Ben-gang Wang, Department 1 of General Surgery, the First Hospital of China Medical University, North Nanjing Street 155#, Heping District, Shenyang110001, China. Telephone：+86-024-83283308; fax: +86-024-83282383. Email：bgwang@cmu.edu.cn.

<Contents>

Supplementary Table 1. The captured SNPs covered by the selected tagSNPs

Supplementary Table 2. The baseline of the subjects

Supplementary Figure 1. The linkage disequilibrium (LD) of *PCAT1* gene

Supplementary Table 1. The captured SNPs covered by the selected tagSNPs

TagSNPs	Captured SNPs
rs4573233	rs10481163,rs17762938,rs4473999,rs1551515,rs2124597,rs10481164,rs73351641, rs7824393,rs7823764,rs6651240,rs16901904,rs7842175,rs10109680,rs1551513, rs9656964,rs7823297,rs7824011,rs7463326,rs17762878,rs4573233
rs710885	rs785004,rs2632160,rs785003,rs710885,rs785002
rs1902432	rs1026411,rs1902432,rs1551514,rs2015595
rs785005	rs785005,rs2676456,rs2606181
rs2632159	rs2632159,rs931354

Supplementary Table 2. The baseline of the subjects

Variables	*P*		CRC		
	CRC vs. CON	CON (%)	All (%)	Colon Cancer (%)	Rectal Cancer (%)
		**n=510**	**n=436**	**n=229**	**n=207**
Gender					
Male		234(45.9)	252(57.8)	120(52.4)	132(63.8)
Female	＜0.001	276(54.1)	184(42.2)	109(47.6)	75(36.2)
Age					
Mean±SD		49.9±15.0	59.6±10.2	59.9±10.8	59.2±9.54
Median		49	60	61	60
Range	＜0.001	23-90	22-82	24-82	22-78

Note: CON, controls; CRC, colorectal cancer.
